# To be born twin: effects on long-term neurodevelopment of very preterm infants—a cohort study

**DOI:** 10.3389/fped.2023.1217650

**Published:** 2023-07-17

**Authors:** Camilla Fontana, Paola Schiavolin, Giulia Ardemani, Danila Angela Amerotti, Nicola Pesenti, Chiara Bonfanti, Tiziana Boggini, Silvana Gangi, Matteo Porro, Chiara Squarza, Maria Lorella Giannì, Nicola Persico, Fabio Mosca, Monica Fumagalli

**Affiliations:** ^1^ Fondazione IRCCS Ca' Granda Ospedale Maggiore Policlinico, NICU, Milan, Italy; ^2^ Department of Clinical Sciences and Community Health, University of Milan, Milan, Italy; ^3^ Department of Statistics and Quantitative Methods, Division of Biostatistics, Epidemiology and Public Health, University of Milano-Bicocca, Milan, Italy; ^4^ Fondazione IRCCS Ca' Granda Ospedale Maggiore Policlinico, Pediatric Physical Medicine and Rehabilitation Service, Milan, Italy; ^5^ Fondazione IRCCS Ca' Granda Ospedale Maggiore Policlinico, Prenatal Diagnosis and Fetal Surgery Unit, Milan, Italy

**Keywords:** twins, monochorionic, dichorionic, prematurity, neurodevelopmental outcome

## Abstract

**Objective:**

To examine the effect of twin birth on long-term neurodevelopmental outcomes in a cohort of Italian preterm infants with very low birth weight.

**Study design:**

We performed a retrospective cohort study on children born in a tertiary care centre. We included children born between 1 January 2007 and 31 December 2013 with a gestational age (GA) of ≤32 weeks and birth weight of <1,500 g. The infants born from twin pregnancies complicated by twin-to-twin transfusion syndrome and from higher-order multiple pregnancies were excluded. The children were evaluated both at 2 years corrected age and 5 years chronological age with Griffiths mental development scales revised (GMDS-R). The linear mixed effects models were used to study the effect of being a twin vs. being a singleton on GMDS-R scores, adjusting for GA, being born small for gestational age, sex, length of NICU stay, socio-economic status, and comorbidity score (CS) calculated as the sum of the weights associated with each of the major morbidities of the infants.

**Results:**

A total of 301 children were included in the study, of which 189 (62.8%) were singletons and 112 (37.2%) were twins; 23 out of 112 twins were monochorionic (MC). No statistically significant differences were observed between twins and singletons in terms of mean general quotient and subscales at both 2 and 5 years. No effect of chorionicity was found when comparing scores of MC and dichorionic twins vs. singletons; however, after adjusting for the CS, the MC twins showed lower scores in the hearing and language and performance subscales at 5 years.

**Conclusion:**

Overall, in our cohort of children born very preterm, twin infants were not at higher risk of neurodevelopmental impairment compared with singletons at pre-school age.

## Introduction

The rate of twin births has increased in the recent four decades across many high-income countries; this is mainly due to the augmented maternal age at childbearing and the increase of medically assisted reproduction (1, 2). According to national estimates, twin and multiple deliveries accounted for 1.5% of all deliveries and 9.6% of births from medically assisted pregnancies in 2021 (3). Compared with singletons, twins have an augmented risk of being born preterm (<37 weeks of gestational age (GA) (in Italy, it has been reported that more than 50% of twins or multiples are born preterm) (4) and have up to a 12-fold risk of very preterm (VPT) birth (<32 weeks of GA) (5). Among all preterm infants, 15%–20% are twins or multiples (6).

Preterm birth is associated with long-term neurodevelopmental impairments, with the youngest infants bearing the highest risk, even in the absence of overt brain damage in conventional neuroimaging studies (7, 8). Despite the improvement in the perinatal management of these fragile patients, the children born very preterm and very low birth weight (VLBW) are at risk of motor and cognitive delays (prevalence estimated to be 16.9% and 20.6%, respectively) and approximately 4.3% of these children develop cerebral palsy (CP) (9). The pathogenesis of developmental disorders is multifactorial and includes several dysmaturational events observed in the brains of preterm infants (10). The major risks for negative deviations of neurodevelopmental trajectories have been attributed to demographic factors, such as male sex, ethnicity, and lower level of parental education (11), as well as to the severity of postnatal morbidities, such as bronchopulmonary dysplasia (BPD), retinopathy of prematurity (ROP), sepsis and necrotising enterocolitis (NEC), intraventricular haemorrhage (IVH), and surgical diseases (12–14).

Within this framework, twin gestation has been traditionally associated with an increased risk of perinatal mortality and morbidity (15, 16). Moreover, it has been suggested that twins are at greater risk of adverse long-term neurodevelopmental outcomes than singletons, but the evidence is inconsistent (17). Moreover, longitudinal studies examining the neurodevelopmental outcomes among preterm twins and higher-order multiples are limited, and few of those studies had long follow-up durations. It remains unclear whether twin pregnancy itself is associated with an increased likelihood of adverse neurodevelopmental outcomes or if this effect is related to twin-induced prematurity. Notably, the disadvantages experienced by twins are mainly attributable to the risk associated with shorter gestational age and poor foetal growth (18). Recent studies (19, 20) have compared the neurodevelopmental outcomes of preterm multiples and preterm singletons and found no differences at a general level while a slightly lower mean score was observed for the locomotor and personal-social subscales (19) and for the verbal intelligence quotient (20).

Chorionicity may play an important role in the neurodevelopmental outcome of twins. Monochorionic (MC) twins share a single placenta with vascular anastomosis connecting the two foetal umbilical circulations and creating haemodynamic instability (21). MC gestation carries a higher risk for complications such as twin-to-twin transfusion syndrome (TTTS), which affects approximately 10%–15% of all MC twin pregnancies (22), selective intrauterine growth restriction (IUGR), and intrauterine death (IUD) of a co-twin (23–25). Interestingly, even in the absence of TTTS, MC twins, especially those born prematurely, seem to be at higher risk of morbidity than preterm dichorionic (DC) twins (26). Several studies have investigated the association between chorionicity and neurodevelopmental outcome, but the findings are controversial (16, 20).

The aim of the present study was to compare the neurodevelopmental outcomes at 2 and 5 years of age of singletons vs. uncomplicated twins from a cohort of premature infants with GA ≤32 weeks and birth weight (BW) <1,500 g. Furthermore, we explored the associations between different chorionicity features and neurodevelopmental outcomes.

## Materials and methods

We performed a retrospective cohort study. The children were considered eligible if they were born between 1 January 2007 and 31 December 2013 at the Tertiary Neonatal Intensive Care Unit of Fondazione IRCCS Ca’ Granda Ospedale Maggiore Policlinico in Milan, Italy, with GA ≤32 weeks and BW <1,500 g and were admitted to the NICU within 6 h of life. The exclusion criteria were major congenital anomalies, metabolic and genetic syndromes, death before NICU discharge, transfer to another NICU within the first 24 h of life, infants born as singletons after intrauterine death of one or more foetuses, twin pregnancy complicated by TTTS, and higher-order multiple pregnancies. Only the children who underwent neurodevelopmental assessment at both 24 ± 6 months of corrected age and 5 ± 1 years of chronological age were included in the analysis. This study is a part of a larger cohort study evaluating the effects of very premature birth and related complications on long-term neurodevelopmental outcomes; the results regarding the effects of red blood cell transfusion have already been published (27). The cohort included in the present analysis partially overlaps with a previously published cohort (including all infants born between 2007 and 2011 at the same Institution independently from GA at birth) reporting only the data on early neonatal outcomes (28).

The neurodevelopment of the infants was assessed at the Paediatric Physical Medicine and Rehabilitation Unit of the same Foundation according to the local follow-up programme for preterm infants. Approval from the Ethical Committee of Foundation IRCCS Ca’ Granda Area 2 was obtained. Due to the retrospective nature of the study and the anonymisation of all the data collected, which are presented as aggregate data, the requirement for informed consent was waived.

### Data collection and definitions

The twins were classified as MC or DC twins according to chorionicity assessed by the first-trimester ultrasound scan (29, 30). The perinatal data, postnatal morbidities, treatments, and sociodemographic data from birth to discharge were collected from the electronic medical chart review. Small for gestational age (SGA) was defined as birth weight <10th percentile for GA (31), and the clinical risk index for babies (CRIB) II was calculated based on GA, BW, sex, temperature, and base excess at birth (32). Sepsis was defined by positive blood culture (33) with clinical signs of infection, BPD as requiring oxygen at 36 weeks of postmenstrual age (34). NEC was classified according to the modified Bell's staging criteria (35) and ROP according to the International Classification of ROP (36). Cerebral lesions as detected by cranial ultrasound were classified as follows: IVH grades according to Papile's classification (37), cystic periventricular leukomalacia (cPVL), cerebral focal lesion (defined as any other focal grey and white matter abnormalities as detected by cranial ultrasound), posthaemorrhagic ventricular dilatation (PHVD), cerebellar haemorrhage detectable by cranial ultrasound (>4 mm in size), and major cerebral malformations. Socio-economic status (SES) was calculated with the Hollingshead four-factor index (38).

### Neurodevelopmental outcomes

The follow-up data were retrieved from the clinical files of the infants. Neurodevelopmental assessments at 24 ± 6 months of corrected age and 5 ± 1 years of chronological age were assessed using the Griffiths mental development scales revised (GMDS-R) (39) and extended revised (GMDS-ER) (40) for ages 0–2 and 2–8 years, respectively, and were performed by trained developmental specialists who were blinded to the clinical histories of the infants but were aware of the child being a twin and the type of chorionicity. The Griffiths scales consist of 5 subscales (6 for the extended version): locomotor, personal-social, hearing and language, eye and hand coordination, performance, and practical reasoning (only for the extended version). The locomotor subscale evaluates gross motor skills. The personal-social subscale assesses the level of independency in daily living activities and social development. The hearing and language subscale investigates both receptive and expressive language. The eye and hand coordination subscale measures fine motor skills, visual monitoring skills, and manual dexterity. The performance subscale investigates the ability to reason through performance tests, mainly focusing on visuospatial skills. The practical reasoning subscale covers a range of reasoning skills, including learning numerical concepts and time and space orientation. A composite general quotient [GQ, mean 100, standard deviation (SD) 12] and separate standardised quotients for each subscale (mean = 100, SD = 16) can be calculated. Accordingly, the children were classified as having the following: typical development if their GQ was 88 or higher and their subquotients were 84 or higher; a developmental delay if their GQ was lower than 88 and their subquotients were lower than 84. Because the normative data of the GMDS-R are not available in our country, we referred to the 1996 United Kingdom norms (41).

### Statistical analyses

The statistical analyses were performed using R Software (R Foundation for Statistical Computing, Vienna, Austria) (42). The descriptive statistics of twins and singletons are presented. The continuous variables following normal or non-normal distribution were reported as the mean (SD) or median (IQR) and compared among groups with the Student's *t*-test or Mann–Whitney *U* test. The categorical variables are presented as the absolute frequency (percentage) and were compared with *χ*^2^ test or Fisher's exact test (when some of the cells had counts fewer than 5). To control for between-family variability, the linear mixed effects models were used to study the effect of being a twin vs. being a singleton on GMDS-R at 2 and 5 years of age, and the family was considered a random effect. The models were adjusted for GA, SGA, sex, length of NICU stay (LoS), SES, and comorbidity score (CS).

The CS (43) was calculated as the sum of the weights associated with each of the major morbidities of the infants: severe BPD, sepsis, ROP grade 3–4, NEC (both medical and surgical), severe brain injury (IVH grade 3 or 4, cPVL, PHVD, cerebellar haemorrhage or cerebral focal lesions), and need for major surgery. The weights were determined using a linear regression model for each outcome of interest (dependent variables) and comorbidities as independent variables. The effect of being MC or DC twins vs. being singletons on GMDS-R at 2 and 5 years of age was also studied.

All tests were two-tailed, and a *p*-value <0.05 was considered significant.

## Results

**Figure 1:**
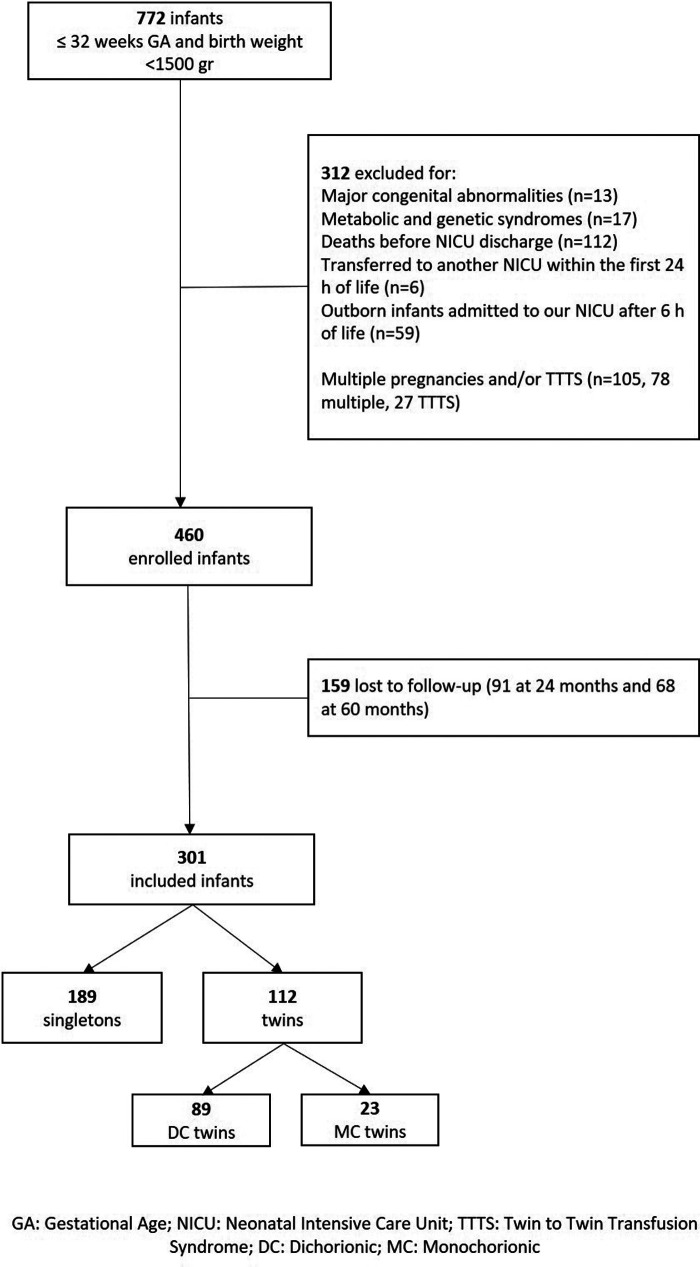
Participant flowchart.

During the study period, a total of 772 infants were born with GA ≤32 weeks and birth weight <1,500 g. A total of 312 infants were excluded due to predefined criteria (Figure 1). A total of 460 preterm infants were enrolled in the larger cohort study; 159 out of 460 were lost to follow-up (91 at 24 months and 68 at 60 months). A total of 301 infants were eligible for this part of the study, including 189 (62.8%) singletons and 112 (37.2%) twins; 23 out of 112 twins (20.5%) were MC. The flowchart of the study is reported in Figure 1. The clinical characteristics of the included infants of this part of the study are presented in Tables 1–3.

**Table 1:** Descriptive comparisons between singletons and twins.

Variables	Singletons (*n* = 189)	Twins (*n* = 112)	*p*-value*
Gestational age at birth (weeks), mean (SD)	28.6 (2.3)	29.2 (2.4)	**0.039**
Birth weight (g), mean (SD)	1,033.8 (288.2)	1,157.5 (285.7)	**<0** **.** **001**
Small for gestational age, *n* (%)	69 (36.5)	24 (21.4)	**0** **.** **009**
Male gender, *n* (%)	97 (51.3)	45 (40.2)	0.08
Caesarean section, *n* (%)	170 (89.9)	109 (97.3)	**0** **.** **032**
Apgar 1′, median (IQR)	6 (5–8)	7 (6–8)	**0** **.** **014**
Apgar 5′, median (IQR)	8 (8–9)	9 (8–9)	**0** **.** **006**
CRIB score, mean (SD)	8.4 (3.5)	6.9 (3.7)	**0** **.** **003**
Invasive ventilation (<14 days of life) (days), mean (SD)	7.77 (14.9)	6.7 (14.1)	0.54
Red blood cell transfusion number, median (IQR)	1 (0–4)	0 (0–2)	0.068
Cardiovascular drug support, *n* (%)	50 (26.5)	31 (27.7)	0.923
Sepsis (early and late), *n* (%)	77 (40.7)	35 (31.2)	0.128
Surgical NEC, *n* (%)	6 (3.2)	6 (5.4)	0.373
Surgical PDA, *n* (%)	10 (5.3)	5 (4.5)	0.964
Severe ROP (grade 3–4), *n* (%)	17 (9.0)	10 (8.9)	>0.999
Severe BPD (grade 3), *n* (%)	23 (12.2)	13 (11.6)	>0.999
IVH grade 3 or 4, *n* (%)	6 (3.2)	5 (4.5)	0.545
Posthaemorrhagic ventricular dilatation, *n* (%)	4 (2.1)	1 (0.9)	0.737
Cystic periventricular leukomalacia, *n* (%)	1 (0.5)	2 (1.8)	0.645
Cerebral focal lesion, *n* (%)	2 (1.1)	0 (0.0)	0.72
Cerebellar haemorrhage, *n* (%)	2 (1.1)	3 (2.7)	0.551
Length of stay (days), median (IQR)	68 (50–95)	57 (41–81)	0.009
Maternal age at birth (years), mean (SD)	34.6 (5.5)	35.9 (5.7)	0.057
Socio-economic status, mean (SD)	36.9 (15.6)	39.9 (12.4)	0.083

SD, standard deviation; IQR, interquartile range; CRIB II, clinical risk index for babies score; NEC, necrotising enterocolitis; PDA, patent ductus arteriosus; ROP, retinopathy of prematurity; BPD, bronchopulmonary dysplasia; IVH, intraventricular haemorrhage.

Data in bold are the significant ones.

* *p*-values from *t*-test or Mann–Whitney *U* test (continuous variables); *χ*^2^ test or Fisher's exact test (categorical variables).

**Table 2:** Descriptive comparisons between singletons and MC twins.

Variables	Singletons (*n* = 189)	MC twins (*n* = 23)	*p*-value*
Gestational age at birth (weeks), mean (SD)	28.6 (2.3)	30.0 (1.9)	**0** **.** **009**
Birth weight (g), mean (SD)	1,033.8 (288.2)	1,281.5 (251.6)	**<0** **.** **001**
Small for gestational age, *n* (%)	69 (36.5)	6 (26.1)	0.45
Male gender, *n* (%)	97 (51.3)	5 (21.7)	**0** **.** **014**
Caesarean section, *n* (%)	170 (89.9)	23 (100.0)	0.227
Apgar 1′, median (IQR)	6 (5–8)	7 (6–8)	**0.014**
Apgar 5′, median (IQR)	8 (8–9)	9 (8–9)	0.055
CRIB score, mean (SD)	8.4 (3.5)	5.5 (3.2)	**0** **.** **001**
Invasive ventilation (<14 days of life) (days), mean (SD)	7.77 (14.9)	4.1 (10.0)	0.25
Red blood cell transfusion number, median (IQR)	1 (0–4)	0 (0–0.5)	**0** **.** **008**
Cardiovascular drug support, *n* (%)	50 (26.5)	7 (30.4)	0.875
Sepsis (early and late), *n* (%)	77 (40.7)	4 (17.4)	0.051
Surgical NEC, *n* (%)	6 (3.2)	0 (0.0)	>0.999
Surgical PDA, *n* (%)	10 (5.3)	1 (4.3)	>0.999
Severe ROP (grade 3–4), *n* (%)	17 (9.0)	1 (4.3)	0.72
Severe BPD (grade 3), *n* (%)	23 (12.2)	2 (8.7)	0.884
IVH grade 3 or 4, *n* (%)	6 (3.2)	1 (4.3)	0.558
Posthaemorrhagic ventricular dilatation, *n* (%)	4 (2.1)	0 (0.0)	>0.999
Cystic periventricular leukomalacia, *n* (%)	1 (0.5)	0 (0.0)	>0.999
Cerebral focal lesion, *n* (%)	2 (1.1)	0 (0.0)	>0.999
Cerebellar haemorrhage, *n* (%)	2 (1.1)	1 (4.3)	0.744
Length of stay (days), median (IQR)	68 (50–95)	52 (34–59)	**0** **.** **001**
Maternal age at birth (years), mean (SD)	34. 6 (5.5)	34.9 (7.1)	0.779
Socio-economic status, mean (SD)	36.9 (15.6)	34.6 (14.0)	0.505

SD, standard deviation; IQR, interquartile range; CRIB II, clinical risk index for babies score; NEC, necrotising enterocolitis; PDA, patent ductus arteriosus; ROP, retinopathy of prematurity; BPD, bronchopulmonary dysplasia; IVH, intraventricular haemorrhage; MC, monochorionic.

Data in bold are the significant ones.

* *p*-values from *t*-test or Mann–Whitney *U* test (continuous variables); *χ*^2^ test or Fisher's exact test (categorical variables).

**Table 3:** Descriptive comparisons between singletons and DC twins.

Variables	Singletons (*n* = 189)	DC twins (*n* = 89)	*p*-value*
Gestational age at birth (weeks), mean (SD)	28.6 (2.3)	29.0 (2.5)	0.205
Birth weight (g), mean (SD)	1,033.8 (288.2)	1,125.5 (286.5)	**0** **.** **014**
Small for gestational age, *n* (%)	69 (36.5)	18 (20.2)	**0** **.** **010**
Male gender, *n* (%)	97 (51.3)	40 (44.9)	0.388
Caesarean Section, *n* (%)	170 (89.9)	86 (96.6)	0.092
Apgar 1′, median (IQR)	6 (5–8)	7 (6–8)	0.075
Apgar 5′, median (IQR)	8 (8–9)	9 (8–9)	**0** **.** **019**
CRIB score, mean (SD)	8.4 (3.5)	7.3 (3.8)	**0** **.** **047**
Invasive ventilation (<14 days of life) (days), mean (SD)	7.77 (14.9)	7.9 (14.9)	0.839
Red blood cell transfusion number, median (IQR)	1 (0–4)	0 (0–3)	0.346
Cardiovascular drug support, *n* (%)	50 (26.5)	24 (27.0)	>0.999
Sepsis (early and late), *n* (%)	77 (40.7)	31 (34.8)	0.417
Surgical NEC, *n* (%)	6 (3.2)	6 (6.7)	0.208
Surgical PDA, *n* (%)	10 (5.3)	4 (4.5)	>0.999
Severe ROP (grade 3–4), *n* (%)	17 (9.0)	9 (10.1)	0.938
Severe BPD (grade 3), *n* (%)	23 (12.2)	11 (12.4)	>0.999
IVH grade 3 or4, *n* (%)	6 (3.2)	4 (4.5)	0.731
Posthaemorrhagic ventricular dilatation, *n* (%)	4 (2.1)	1 (1.1)	0.922
Cystic periventricular leukomalacia, *n* (%)	1 (0.5)	2 (2.2)	0.502
Cerebral focal lesion, *n* (%)	2 (1.1)	0 (0.0)	0.831
Cerebellar haemorrhage, *n* (%)	2 (1.1)	2 (2.2)	0.813
Length of stay (days), median (IQR)	68 (50–95)	60 (42–100)	0.120
Maternal age at birth (years), mean (SD)	34. 6 (5.53)	36.1 (5.3)	**0** **.** **031**
Socio-economic status, mean (SD)	36.9 (15.55)	41.3 (11.6)	**0** **.** **019**

SD, standard deviation; IQR, interquartile range; CRIB II, clinical risk index for babies score; NEC, necrotising enterocolitis; PDA, patent ductus arteriosus; ROP, retinopathy of prematurity; BPD, bronchopulmonary dysplasia; IVH, intraventricular haemorrhage; DC, dichorionic.

Data in bold are the significant ones.

* *p*-values from *t*-test or Mann–Whitney *U* test (continuous variables); *χ*^2^ test or Fisher's exact test (categorical variables).

### Neurodevelopmental outcomes: singletons vs. twins

The mean GMDS-R GQ was similar in singletons and twins at both 24 ± 6 months of corrected age (singletons = 89.8, SD = 15.0 and twins = 88.4, SD = 13.4; *p* = 0.421) and 5 ± 1 years (singletons = 88.4, SD = 11.2 and twins = 88.4, SD = 10.9; *p* = 0.998). No significant differences were observed between singletons and twins in the GMDS-R subquotients (Table 4). In the model adjusted for GA, SGA, sex, LoS, SES, and comorbidity score, no differences emerged between the two groups at both short- and long-term neurodevelopmental assessments (Table 4).

**Table 4:** Griffiths scores at 24 ± 6 months and at 5 ± 1 years expressed as mean (SD) for singletons vs. twins.

		Singletons	Twins	*p*-value*	*B*	*p*-value
24 months	General quotient	89.8 (15.0)	88.4 (13.43)	0.421	−1.87	0.251
	Locomotor	90.0 (17.5)	91.6 (15.2)	0.415	0.6	0.748
	Personal-social	84.5 (16.0)	85.0 (13.7)	0.775	−0.48	0.796
	Hearing and speech	89.3 (17.0)	86.7 (15.3)	0.178	−2.58	0.198
	Eye and hand coordination	94.5 (16.1)	91.9 (13.1)	0.142	−2.84	0.115
	Performance	92.3 (15.5)	91.0 (13.2)	0.435	−2.61	0.129
5 years	General quotient	88.4 (11.2)	88.4 (11.0)	0.998	−0.73	0.529
	Locomotor	91.0 (11.7)	90.9 (12.4)	0.979	−0.32	0.790
	Personal-social	88.7 (10.3)	87.9 (11.0)	0.482	−1.52	0.208
	Hearing and speech	89.7 (12.3)	88.5 (12.3)	0.428	−1.89	0.195
	Eye and hand coordination	86.9 (11.7)	87.2 (12.6)	0.800	−0.43	0.742
	Performance	88.8 (10.7)	88.4 (12.2)	0.753	−1.17	0.334
	Practical reasoning	88.0 (12.0)	88.2 (11.6)	0.871	−0.81	0.550

* *p*-values from *t*-test or Mann–Whitney *U* test (continuous variables); *χ*^2^ test or Fisher's exact test (categorical variables).

*B* represents the linear mixed effect model estimate, along with the *p*-value, of being twin compared with singleton adjusted for GA, SGA, sex, LoS, SES, and comorbidity score.

### Neurodevelopmental outcomes: singletons vs. twins according to chorionicity

The mean GMDS-R GQ was similar in singletons and MC twins at both 24 ± 6 months of corrected age (singletons = 89.8, SD = 15 and MC twins = 88.9, SD = 16.4; *p* = 0.778) and 5 ± 1 years (singletons = 88.4, SD = 11.2 and MC twins = 86.7, SD = 10.9; *p* = 0.507). No significant differences were observed between MC twins and singletons in the GMDS-R subquotients when not corrected for the main clinical characteristics (Table 5). However, after adjusting for GA, SGA, sex, LoS, SES, and comorbidity score, a significant difference between the two groups emerged at the assessment after 5 ± 1 years in the hearing and language and in the performance subscales (Table 5), with MC twins showing lower scores. No differences between DC twins vs. singletons were observed at 24 months and 5 years, in GQ and subscales, even after adjustment (Table 6).

**Table 5:** Griffiths scores at 24 ± 6 months and at 5 ± 1 years expressed as mean (SD) for singletons vs. MC.

		Singletons	MC twins	*p*-value*	B	*p*-value
24 months	General quotient	89.8 (15.0)	88.9 (16.4)	0.778	−2.49	0.390
	Locomotor	90.0 (17.5)	93.6 (16.1)	0.366	0.28	0.943
	Personal-social	84.5 (16.0)	86.5 (17.3)	0.582	−0.54	0.875
	Hearing and speech	89.3 (17.0)	88.0 (17.8)	0.736	−2.36	0.511
	Eye and hand coordination	94.5 (16.1)	89.6 (17.8)	0.183	−5.7	0.097
	Performance	92.3 (15.5)	87.4 (17.1)	0.162	−5.96	0.072
5 years	General quotient	88.4 (11.2)	86.7 (10.9)	0.507	−4.2	0.072
	Locomotor	91.0 (11.7)	91.6 (10.8)	0.809	−1.54	0.549
	Personal-social	88.7 (10.3)	86.5 (10.9)	0.330	−4.13	0.086
	Hearing and speech	89.7 (12.3)	86.1 (14.1)	0.202	−5.93	**0** **.** **040**
	Eye and hand coordination	86.9 (11.7)	87.4 (10.9)	0.853	−2.11	0.412
	Performance	88.8 (10.7)	85.6 (12.4)	0.194	−5.15	**0** **.** **016**
	Practical reasoning	88.0 (12.0)	85.9 (13.3)	0.446	−4.55	0.087

Data in bold are the significant ones.

* *p*-values from *t*-test or Mann–Whitney *U* test (continuous variables); *χ*^2^ test or Fisher's exact test (categorical variables).

*B* represents the linear mixed effect model estimate, along with the *p*-value, of being monochorionic compared with singleton adjusted for GA, SGA, sex, LoS, SES, and CS.

**Table 6:** Griffiths scores at 24 ± 6 months and at 5 ± 1 years expressed as mean (SD) for singletons vs. DC.

		Singletons	DC twins	*p*-value*	*B*	*p*-value
24 months	General quotient	89.8 (15.0)	88.3 (12.7)	0.415	−1.7	0.336
	Locomotor	90.0 (17.5)	91.2 (15.0)	0.593	0.1	0.954
	Personal-social	84.5 (16.0)	84.6 (12.8)	0.936	−0.6	0.763
	Hearing and speech	89.3 (17.0)	86.3 (14.7)	0.156	−2.4	0.269
	Eye and hand coordination	94.5 (16.1)	92.4 (11.7)	0.272	−2.3	0.232
	Performance	92.3 (15.5)	91.8 (12.1)	0.794	−2.0	0.276
5 years	General quotient	88.4 (11.2)	88.8 (10.9)	0.765	0.0	0.984
	Locomotor	91.0 (11.7)	90.8 (12.9)	0.896	−0.1	0.948
	Personal-social	88.7 (10.3)	88.2 (11.0)	0.687	−1.0	0.449
	Hearing and speech	89.7 (12.3)	89.1 (11.9)	0.716	−1.1	0.464
	Eye and hand coordination	86.9 (11.7)	87.2 (13.1)	0.830	−0.1	0.959
	Performance	88.8 (10.7)	89.1 (12.1)	0.858	−0.1	0.952
	Practical reasoning	88.0 (12.0)	88.8 (11.2)	0.596	0.1	0.926

* *p*-values from *t*-test or Mann–Whitney *U* test (continuous variables); *χ*^2^ test or Fisher's exact test (categorical variables).

*B* represents the linear mixed effect model estimate, along with the *p*-value, of not being monochorionic compared with singleton adjusted for GA, SGA, sex, LoS, SES, and CS.

## Discussion

The impact of twin birth on the long-term neurodevelopment of prematurely born infants is still a matter of debate. Our findings show that preterm twins have similar neurodevelopment at 2 and 5 years of age compared with singleton preterm-born children, supporting the hypothesis that being born twin is not an additional risk factor for neurodevelopmental impairment and that the neurodevelopmental outcomes are mainly related to prematurity itself and consequential morbidities.

The available evidence on this topic is controversial, and previous studies have reported contrasting results. Consistent with our findings, Gnanendran et al. (44) observed that the neurodevelopmental outcomes at 2–3 years of multiples (twins, triplets, quadruplets) compared with singleton extremely preterm infants <29 weeks GA were similar. Ylijoki et al. (20) evaluated cognitive development, neuropsychological performance, and neurodevelopmental impairments at 5 years in a population of VLBW and VPT infants born singleton and twins and concluded that twins had similar neuropsychological outcomes to singletons, despite having a slightly lower verbal intelligence quotient. In contrast, Bodeau-Livinec et al. (45) found that VLBW twins had higher mortality and slightly lower cognitive scores than singletons after adjustment for sex, GA, IUGR, and SES but no difference with respect to severe deficiencies at 5 years of age. Nevertheless, this study included twin pregnancies complicated by TTTS, potentially affecting the results.

In their review, Lorenz (46) highlighted that several older population-based studies reported a higher prevalence of delay in the achievement of cognitive neurodevelopmental milestones among twins compared with singletons, but not all the studies were adjusted for GA and BW, which are two of the most important risk factors for long-term neurodevelopmental impairments (46); furthermore, they did not exclude TTTS. Therefore, we decided to consider GA and BW as part of the inclusion criteria to compare two homogeneous groups of infants. The neurodevelopment of preterm infants is affected by a dense interplay of causal factors. In our population, singletons had lower GA (mean = 28.6, SD = 2.3 weeks) than twins (mean = 29.2, SD = 2.3 weeks), were more likely to be born SGA (36.5% of singletons vs. 20.8% of twins) and male (51.3% vs. 39.0%), had a higher CRIB score (mean = 8.4, SD = 3.5 vs. 6.9 SD = 3.7), and had a longer LoS (median 68 vs. 56 days). Despite these covariates, we did not observe any differences in the neurodevelopmental outcome at 2 and 5 years of age between singletons and twins, even after adjusting for the main confounding factors.

Interestingly, our data concerning perinatal characteristics of singletons and twins are somewhat in contrast with the literature. Kalikkot Thekkeveedu et al. (47) found the highest percentage of premature birth among twins compared with singletons, with twins having a lower mean GA at birth. Moreover, in their study, the number of SGA neonates was significantly higher among twins than among singletons, whereas the median LoS was significantly shorter for singleton within each GA category. Our hospital is one of the national referral centres for high-risk pregnancies, and singletons were more likely to be born prematurely due to an iatrogenically induced preterm delivery because of maternal or foetal diseases (maternal hypertension, pre/eclampsia, foetal growth restriction) rather than spontaneous preterm labour *per se*, resulting in sicker preterm infants at birth. These observations may at least partially explain the differences compared with the other studies; unfortunately, due to the retrospective nature of our study, the data regarding maternal or obstetric complications were not available to support this hypothesis.

Finally, in the review of Babatunde et al. (17) on neurodevelopment differences between twins and singletons, including the studies published between 2011 and 2017, five of the eight articles showed no significant difference in the neurodevelopmental outcomes between twins and singletons while two showed that singletons had better academic outcomes than twins. However, they did not conduct a meta-analysis due to the low number of the studies included and the heterogenicity of the study populations and instruments to measure the neurodevelopmental outcomes.

We decided to further investigate the impact of twin birth by considering the role of chorionicity as a risk factor for adverse neurodevelopmental outcomes. We *a priori* decided to exclude MC pregnancies complicated by TTTS, which represents a well-known neurological risk factor, with both donor and recipient survivors at risk for antenatally acquired brain damage. A higher risk of neurological morbidities (26) in MC pregnancies has also been reported in cases of single IUD and selective IUGR (48). However, even in the absence of these complications, the typical placental architecture in MC pregnancies may lead to a transitory cardiovascular imbalance responsible for cerebral hypoperfusion and subsequent brain damage (26, 49). Nonetheless, evidence is still scarce, as few studies have focused on twin pregnancies without complication. In our study, despite the overall similarity with singletons, after adjusting for the major comorbidities, uncomplicated MC twins at 5 years revealed a less favourable neurodevelopmental outcome in the hearing and speech and performance GMDS-ER subscales, compared with singletons; conversely, no differences emerged in the DC population compared with singletons.

Several studies previously compared MC twins with DC twins but not with singletons. Hack et al. (50) did not observe any differences in developmental outcome between uncomplicated MC infants and DC infants at 22 months of corrected age, except for a higher incidence of mild delay in hearing and language in uncomplicated MC infants. However, in this study, the mean GA of the population was higher (approximately 35 weeks) than that of our population. Similarly, Ichinomiya et al. (16) described a significant verbal disability and social impairment at 3 years in VLBW MC twins vs. VLBW DC twins, assuming that the cerebral areas associated with verbal and language functions may be vulnerable to suboptimal placental flows that are typical of the MC pregnancies. However, this difference was not observed when only MC twins without TTTS were considered. In contrast, Adegbite et al. (51) found that neurologic morbidity in MC infants at 2 years was 7-fold higher than that in DC infants; however, in this study, the TTTS, IUD, and discordant birth weight were the main determinants of neurodevelopmental impairments. Finally, in a recent study, Tosello et al. (52) found no differences in the neurodevelopmental outcome between MC, including pregnancies complicated by TTTS, and DC twins less than 34 weeks of GA (incidence of survival without neurosensory impairment >96%).

The major limitations of our work are the small sample of the MC cohort and the retrospective design of the study. The latter limited the possibility of retrieving obstetric variables that could affect the neurodevelopmental outcomes in twin pregnancies, such as information about assisted reproductive technology pregnancy, evidence and severity of placental blood flow alteration, and weight discordance fluctuation between the foetuses. In particular, we were not able to investigate the effect of selective IUGR, which is a known condition potentially affecting the neurodevelopment outcome. In addition, although we adjusted for severe comorbidities and socio-economic status, it was not possible to deepen the analysis of the family condition, as we could not gather information on the presence of other siblings and, more generally, on the home environment, which is known to play a key role in child neurodevelopment (53). Lastly, the developmental specialists performing the follow-up assessment were not blind to whether the child was a twin or a singleton and to the chorionicity of twins.

One of the main strengths of the study was the relatively large cohort of VPT and VLBW infants assessed longitudinally up to 5 years of age. Indeed, the previous studies mainly focused on short-term developmental outcomes (19, 54) that, as observed here, might not be predictive of the long-term neurodevelopmental profile in this group of infants. In addition, all infants were enrolled at the same institute, thereby limiting the confounding factors related to the potential differences in medical assistance among centres.

In conclusion, in our cohort of children born very preterm, twin birth was not associated with an increased risk of neurodevelopmental impairment compared with singletons, both at 24 months and 5 years of age. This confirms that twin birth itself is not an added risk factor for an unfavourable developmental outcome, although uncomplicated MC twins seem to have a somewhat less favourable neurodevelopmental outcome at 5 years of age supporting the need of a close neurodevelopmental follow-up. Although the present study may be useful when counselling the parents of twin foetuses at risk for preterm delivery, it is important to note that the number of infants in our MC group was too small to draw a definite conclusion on this population.

## Data Availability

The raw data supporting the conclusions of this article will be made available by the authors, without undue reservation.
